# Patients Undergoing Systemic Anti-Cancer Therapy Who Require Surgical Intervention: What Surgeons Need to Know

**DOI:** 10.3390/cancers15153781

**Published:** 2023-07-26

**Authors:** Matthew D. Robinson, Mairéad G. McNamara, Hamish W. Clouston, Paul A. Sutton, Richard A. Hubner, Juan W. Valle

**Affiliations:** 1 Division of Cancer Sciences, School of Medical Sciences, Faculty of Biology Medicine and Health, The University of Manchester, Manchester M13 9NT, UK; 2 Department of Medical Oncology, ENETS Centre of Excellence, The Christie NHS Foundation Trust, Wilmslow Road, Manchester M20 4BX, UK; 3 Colorectal and Peritoneal Oncology Centre, The Christie NHS Foundation Trust, Wilmslow Road, Manchester M20 4BX, UK

**Keywords:** systemic anti-cancer therapy, surgical intervention, surgical complications

## Abstract

**Simple Summary:**

As cancer treatment continues to evolve and novel therapeutic agents are introduced, the landscape of associated safety concerns becomes more complex. It is common for patients with cancer to require some level of surgical procedure either as part of their disease management, for emergency treatment or in symptom control. This review aimed to evaluate the available evidence around surgical complications in patients receiving systemic anti-cancer therapy, examine the recommended risk mitigation options and highlight important safety considerations for patients who require surgery whilst receiving cancer treatment.

**Abstract:**

As part of routine cancer care, patients may undergo elective surgery with the aim of long-term cure. Some of these patients will receive systemic anti-cancer therapy (SACT) in the neoadjuvant and adjuvant settings. The majority of patients, usually with locally advanced or metastatic disease, will receive SACT with palliative intent. These treatment options are expanding beyond traditional chemotherapy to include targeted therapies, immunotherapy, hormone therapy, radionuclide therapy and gene therapy. During treatment, some patients will require surgical intervention on an urgent or emergency basis. This narrative review examined the evidence base for SACT-associated surgical risk and the precautions that a surgical team should consider in patients undergoing SACT.

## 1. Introduction

### 1.1. “Chemotherapy” in the 21st Century

**Figure 1:**
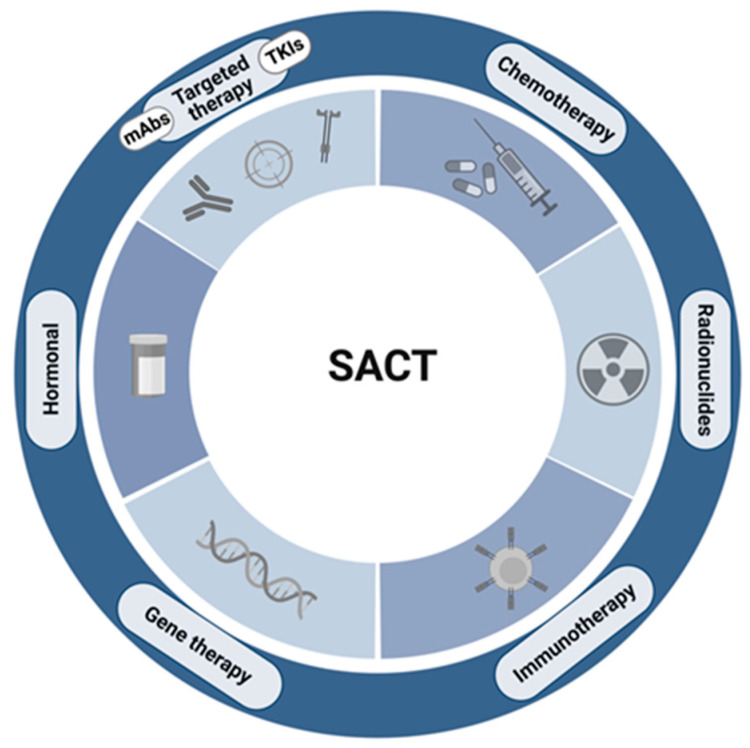
A visual representation of the systemic anti-cancer therapies available in the 21st century. Abbreviations: mAbs; monoclonal antibodies, TKIs; tyrosine kinase inhibitors. Created using Biorender.com.

The landscape of systemic anticancer therapy (SACT) evolved significantly over recent decades due to distillation of the hallmarks of cancer, and improved understanding of the molecular mechanisms driving specific cancer groups, the tumour microenvironment and immune involvement. This progress paved the way towards a more comprehensive treatment armoury that encompasses traditional chemotherapy, targeted therapies, immunotherapy, radionuclides and hormonal therapies (Figure 1). These interventions have been instrumental in facilitating personalised anticancer medicine, led to improved and, in some cases, profound, disease responses and improved toxicity profiles. However, an understanding of the pharmacodynamics of these agents is required by surgeons if the associated adverse effects are to be managed effectively and surgical complication risk mitigated.

### 1.2. A Brief Focus on the Wound-Healing Process

**Figure 2:**
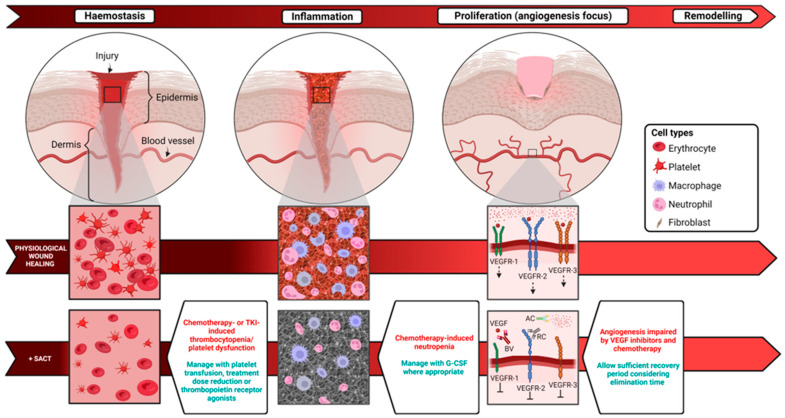
The wound-healing process under physiological condition and the associated changes with SACT administration. The wound-healing cascade consists of a series of regulated processes including haemostasis, inflammation, proliferation and remodelling. Systemic anticancer therapies can impact wound healing at specific stages within the process. Abbreviations: AC, aflibercept; BV, bevacizumab; G-CSF, granulocyte colony stimulating factor; RC, ramucirumab; SACT, systemic anticancer therapy; TKI, tyrosine kinase inhibitor; VEGF, vascular endothelial growth factor; VEGF-R. Created using Biorender.com.

Wound healing is a complex physiological process facilitating the repair of traumatic breaches in the protective barrier. A series of carefully regulated cellular interactions are required across three distinct phases in order to achieve functional restoration of tissue. Haemostasis and inflammation are the initial components of the wound-healing process and incorporate factors that are susceptible to the detrimental effects of SACT on wound healing [1]. The latter stages, proliferation and remodelling are also impacted by components of the anti-cancer armoury [1] (Figure 2).

In order to evaluate the impact of SACT on wound healing, consideration should be given to the cell types involved in the process. There is early involvement of erythrocytes and platelets in the haemostatic component of wound healing [1]. Contact between glycoprotein-VI receptors and endothelial extracellular matrix proteins leads to thrombin amplification and platelet activation [1]. Erythrocyte involvement, either passively via influence on rheology, or actively via biochemical signalling is also crucial for thrombus formation [2,3]. In addition to their role in haemostasis, platelets are integral in immune cell recruitment via eschar entrapment or cytokine-mediated chemotaxis [4]. Neutrophils are also recruited to the site of injury early on and mediate the removal of necrotic tissue and neutralise invading pathogens [5]. Further recruitment of other cell types including fibroblasts and endothelial cells to the site of injury occurs as a result of neutrophil-mediated VEGF and IGF-1 release [6,7].

The initial involvement of erythrocytes and platelets in haemostasis and inflammation suggests that an early clinical focus on full blood count and clotting factor parameters in patients undergoing surgery during, or shortly after, SACT may be beneficial in minimising risk.

In the following section, the relevance of haematological complications of SACT and the associated management strategies will be discussed.

## 2. The Importance of Neutropenia

The haematopoietic toxicities associated with SACT can result in derangement of multiple blood parameters. Perhaps the most clinically significant is absolute neutrophil count (ANC), with chemotherapy-induced neutropenia (CIN) leading to poor patient outcomes, including increased risk of infection-related hospitalisation [8]. Depending on the intervention, cancer type and individual risk factors, incidence of CIN varies between 50.5–87.8% [8,9].

The surgical complications associated with neutropenia can be significant and, in some cases, life-threatening, particularly in the emergent setting. Neutropenia increases the risk of all surgical complications; however, patients with neutropenia are at particularly high risk of developing systemic infections, with mortal potential, due to immunocompromise [10]. There is also the potential for wound-healing complications and neutropenic enterocolitis, among others. The severity of surgical complications in the neutropenic patient, in terms of mortality and morbidity, varies according to aetiology, the nature of the surgical procedure and the surgical setting [11]. The risk of mortality is elevated significantly among patients with complications relating to sepsis, and those undergoing urgent procedures, and is relatively improved in procedures utilising a laparoscopic approach compared with an open approach, for example [11]. Importantly, symptomatology that may usually be considered pathognomonic of infectious or inflammatory pathology, may be diminished in a patient with neutropenia, due to immunocompromise. Therefore, a detailed awareness of CIN risk and the appropriate prevention and management strategies is required if at-risk patients are to be monitored, diagnosed, and managed effectively.

A multicentre, randomised, double-blind, placebo-controlled study investigated the impact of granulocyte colony stimulating factor (G-CSF) as an adjunct to chemotherapy on infection incidence among patients with small-cell lung cancer [12]. Patients were randomised to receive cyclophosphamide, doxorubicin and etoposide either in combination with G-CSF (*n* = 101) or placebo (*n* = 110). The incidence of grade IV neutropenia was significantly lower among those who received G-CSF compared with those who received placebo (*p* = 0.001) [12]. The addition of G-CSF to chemotherapy in this study population was associated with two important observations regarding ANC. Firstly, a dramatic increase in ANC following administration of G-CSF, and secondly, a comparatively accelerated ANC recovery from chemotherapy-induced neutropenia versus placebo [12]. In addition, the median absolute neutrophil count was significantly higher in those randomised to receive G-CSF compared with placebo, at 0.068 × 10^9^/L and 0.036 × 10^9^/L, respectively (*p* = 0.004) [12]. As a consequence of the reduction in extent and duration of neutropenia experienced by participants in the G-CSF group, improvements in clinical outcomes were observed. A reduction in the number of days of antibiotic use and a significantly lower risk of neutropenic fever were associated with G-CSF use compared with those who received placebo (*p* < 0.001) [12]. These observations draw attention to the importance of effective neutropenia management in patients receiving SACT and that appropriate use of G-CSF in the context of primary or secondary prophylaxis correlates with clinically meaningful improvements in patient outcomes.

### Colony Stimulating Factors: The Guidance

The evidence base supporting the use of CSFs to stimulate the production of granulocytes in the management or prevention of neutropenia and the associated life-threatening consequences developed significantly.

Primary prophylaxis involves the administration of CSFs throughout the treatment schedule, including the first cycle. Primary prophylaxis should be utilised in patients with at least a 20% risk of developing febrile neutropenia based on factors relating to the patient, their disease and the treatment [13]. If considered appropriate on a case-specific basis, dose-dense chemotherapy may be an indication for primary prophylaxis with CSFs [13]. Poor nutritional status and ECOG performance status are also indications for G-CSF primary prophylaxis. The American Society of Clinical Oncology (ASCO) recommend consideration of appropriate alternative treatment regimens that do not require CSF support, if available [13].

Secondary prophylaxis with CSFs is recommended in patients who experienced an episode of febrile neutropenia or other neutropenic complication during a previous cycle of treatment where primary prophylaxis was not administered [13].

Dose and treatment duration guidance, for CSFs, varies according to the agent used, the nature of the malignancy and patient demographics. However, G-CSF therapy is often initiated between 24 and 72 h after SACT administration and continued according to the corresponding chemotherapy protocol or until after the ANC nadir and satisfactory ANC restoration [14].

The relevance and role of G-CSF in the context of patients requiring surgical intervention whilst receiving SACT varies according to the urgency of the procedure. In the elective setting, patients with neutropenia may be afforded a sufficient length of time for restoration of the neutrophil count. However, in the emergency setting, G-CSF may be an effective intervention to achieve accelerated recovery of the neutrophil count to mitigate risk of infection as far as possible.

There is an emerging body of evidence, generated predominantly in preclinical models and case reports, supporting the concept of G-CSF-associated tumour progression [15]. Clinical studies aiming to elucidate the relationship between G-CSF and tumour progression and worsened prognosis focus primarily on endogenous G-CSF expression, rather than the use of supportive G-CSF therapy [16]. Further clinical validation of these observations in the prospective setting would be required if treatment guidance is to be adapted to reflect this hypothesis.

## 3. Thrombocytopenia

The implications of thrombocytopenia in patients with cancer can be severe and can range from isolated chemotherapy dose reduction to increased risk of spontaneous bleeding and intra-operative complications.

The incidence of chemotherapy-induced thrombocytopenia is variable according to the specific treatment agent used. In a retrospective study of 614 participants receiving either carboplatin monotherapy or combination therapies including carboplatin, gemcitabine or paclitaxel for the treatment of solid tumours, the incidence and severity of thrombocytopenia was investigated [17]. Overall, thrombocytopenia was observed in 21.8% of patients [17]. However, this was commonly associated with other low blood cell counts including neutropenia or anaemia. Conversely, isolated thrombocytopenia was observed in 6.2% of study participants [17]. In terms of thrombocytopenia severity, grade 3 and 4 thrombocytopenia occurred in 3.6 and 3.3% of participants, respectively, with isolated grade 3 and 4 thrombocytopenia occurring extremely rarely, in 0.7% of participants [17].

Overall, thrombocytopenia occurred in >55% of those who received carboplatin monotherapy or combination regimens including carboplatin, gemcitabine or paclitaxel [17]. Patients receiving gemcitabine in combination with carboplatin or cisplatin, or paclitaxel in combination with carboplatin, were at the highest risk of developing isolated thrombocytopenia [17]. Whilst the focus in this review is on the aforementioned agents, thrombocytopenia is a well-recognised event associated with most chemotherapy regimens and must be borne in mind for any patients receiving chemotherapy.

### 3.1. Clinical Considerations for a Patient with Thrombocytopenia Receiving Chemotherapy

Whilst chemotherapy use is an established aetiology for thrombocytopenia whether by inhibition of platelet production, upregulation of platelet destruction or by increased immune-mediated clearance, alternative causes must be considered [18]. During the clinical assessment of a patient with thrombocytopenia receiving chemotherapy, the clinician must establish the nature of the anti-cancer therapy given and a timeline of administration. This information can be interpreted in combination with what is known about the thrombocyte life cycle and the predicted thrombocyte count trajectory associated with the specific treatment agent. Other important considerations include whether there has been any recent infective pathology, any pre-existing or cancer-associated coagulopathy, if any new medications have been started or recent blood product transfusions given [19].

### 3.2. Management of Thrombocytopenia: Is There a Role for Thrombopoietin Mimetics?

The mainstay of management for patients affected by thrombocytopenia during SACT is treatment dose reduction, treatment delay and platelet transfusion.

Guidance relating to the use of platelet transfusion in patients with cancer has been published by multiple oncologic and haematological bodies internationally. The American Society of Clinical Oncology recommends prophylactic platelet transfusion in patients with thrombocytopenia caused by bone marrow dysfunction with a threshold for transfusion of <10 × 10^9^/L for both haematological and solid malignancies [20]. For patients undergoing surgical intervention, this threshold is adjusted to 40 × 10^9^/L to 50 × 10^9^/L [20].

Initial strategies designed to treat thrombocytopenia via manipulation of the thrombopoietin signalling pathway involved recombinant human thrombopoietin. Whilst these interventions achieved clinically beneficial improvements in thrombocyte count, it was observed that persistent thrombocytopenia occurred in some individuals and, so, development was discontinued [21]. Later, small molecule thrombopoietin receptor agonists, including romiplostim and eltrombopag, were developed.

A systematic review and meta-analysis of 39 eligible studies conducted by Soff et al. investigated the use of thrombopoietic agents in the treatment of chemotherapy-induced thrombocytopenia [22]. The study indicated that although thrombopoietic agents conferred significant platelet count increases in several individual studies, a meta-analysis of efficacy determined that thrombopoietic agents did not significantly decrease incidence of grade 3 or 4 thrombocytopenia, or rates of platelet transfusion, chemotherapy dose reduction, or grade ≥2 bleeding when compared with control groups [22]. Despite an absence of robust evidence of effectiveness, in order to fulfil an unmet clinical need, the National Comprehensive Cancer Network (NCCN) recommends consideration of thrombopoietin receptor agonists, specifically romiplostim, in the treatment of chemotherapy-induced thrombocytopenia [23]. These recommendations are based on prospective data generated in the phase II setting; however, two phase III investigations are currently recruiting (NCT03937154 and NCT03362177). In 3–7% of patients with myelodysplastic syndrome (MDS), romiplostim is associated with progression to acute myeloid leukaemia [24,25]. Therefore, when romiplostim features in the management of patients with comorbid MDS, the treating physician should exercise vigilance and monitor myeloblast parameters, with a view to romiplostim discontinuation on detection of an increased myeloblast count.

## 4. Chemotherapy

It is well established that chemotherapeutic agents are associated with a broad range of adverse effects as a consequence of cytotoxicity. As mentioned previously, haematopoietic toxicities lead to neutropenia, thrombocytopenia and subsequent immune impairment (Figure 2). This, combined with a tendency to inhibit angiogenesis, cell migration and fibroblast proliferation, identifies chemotherapy as an intervention of theoretical concern when evaluating the risk of surgical intervention in patients with cancer.

The mechanism by which chemotherapeutic agents disrupt the wound-healing process is variable, but alkylating agents such as cyclophosphamide disrupt the cell cycle, impair neovascularisation and vasodilation, thus dampening the proliferative phase [26,27].

### 4.1. Neoadjuvant Chemotherapy: Is There Significant Risk?

In the following section, the safety of neoadjuvant chemotherapy, regarding wound-related complications, will be evaluated from two perspectives. Firstly, with respect to superficial wounds and, secondly, in relation to manipulation of the bowel.

The risk of postoperative complications among patients receiving neoadjuvant SACT prior to breast surgery was assessed in a systematic review and meta-analysis of 26 studies. The authors observed no evidence of a significant difference in complication rate between those that received neoadjuvant SACT and those that did not (OR: 1.13, *p* = 0.38) [28]. In this investigation, data were collected in relation to superficial complications involving the skin and subcutaneous fat. Although there was significant heterogeneity between the included studies, this investigation indicates that complications rates in this clinical setting are not increased by the use of neoadjuvant chemotherapy [28]. Importantly, given the nature of this study, confounding factors that may have impacted upon surgical complication rate could not be controlled. Specific attention can be drawn to age and interval between chemotherapy and surgery. It was reported that those receiving neoadjuvant SACT were significantly younger than those who did not. Secondly, the interval between neoadjuvant chemotherapy and surgery was inconsistent between the included studies. In clinical practice, this interval is commonly between 4 and 6 weeks; however, the advantages of longer recovery intervals in terms of complication risk must be balanced against the potentially detrimental impact on survival, as demonstrated in a retrospective review of outcomes among more than 1000 patients with breast cancer [29]. In this study, multivariable analyses indicated that although overall survival (OS), locoregional recurrence-free survival (LRFS) and recurrence-free survival (RFS) were equivalent in patients who underwent surgery at ≤4 weeks, 4–6 weeks or >6 weeks, a sensitivity analysis of the same data indicated that surgery at 8–24 weeks was associated with worse OS and RFS, when compared to surgery at 0–8 weeks after neoadjuvant therapy [29]. It is also important to consider that the optimal interval time may differ or be of less prognostic relevance according to the type of malignancy, as suggested by a meta-analysis including 1171 patients with advanced gastric cancer [30]. In this meta-analysis, no significant difference in pathological complete response rate, R0 resection rate, 3-year PFS or OS, or serious postoperative complication incidence could be identified in relation to time to surgery (<4 week, 4–6 weeks or >6 weeks) [30].

A second systematic review and meta-analysis of seven studies, including over 29,000 patients in total, investigated perioperative complications rates associated with neoadjuvant chemotherapy in patients undergoing surgical intervention for locally advanced cancer of the colon [31]. In an analysis of surgical complications among 430 patients receiving either neoadjuvant (*n* = 238) or adjuvant (*n* = 192) chemotherapy, neoadjuvant chemotherapy was not associated with significantly increased risk of abdominal infection (RR = 1.14, *p* = 0.70) [31]. In the same study, a separate analysis of 1928 patients indicated that risk of anastomotic leakage was not statistically different between those receiving neoadjuvant chemotherapy (*n* = 1084) versus adjuvant chemotherapy (*n* = 844) [31].

Some studies indicated that neoadjuvant chemotherapy, particularly in combination with radiotherapy, is associated with increased surgical complication risk in patients with oesophageal cancer undergoing resection surgery. However, a meta-analysis of 17 randomised controlled trial (RCT)s, including more than 2800 patients, determined that neither neoadjuvant chemotherapy nor chemoradiotherapy were associated with increased risk of anastomotic leakage, a complication of particular concern among surgeons performing oesophageal resection surgery [32]. Other studies have drawn specific attention to increased incidence of pulmonary complications and vocal cord paralysis, although data from a second meta-analysis of 18 studies failed to demonstrate any statistically significant increase in risk for any of the included complications [33].

The aforementioned data go some way in providing reassurance that risk of surgical complications is not significantly increased with neoadjuvant chemotherapy relative to either adjuvant chemotherapy or surgical intervention alone. However, investigations designed in the prospective setting are required to provide assurance and to facilitate the development of guidance regarding interval timing between chemotherapy and surgery.

It is also important to appreciate that surgical risk may differ between patients according to stage of disease or whether SACT is administered as a palliative intervention. Although studies investigating surgical outcomes are seldom conducted in patients with advanced cancer who are receiving palliative SACT, possibly as a consequence of poor performance status precluding trial participation or limiting surgical options, inferences can be made from investigations that include participants with late-stage disease. A retrospective analysis of surgical outcomes in patients with gastric cancer, who received chemotherapy prior to surgery, included tumour stage in multivariate analyses. All 123 participants had gastric adenocarcinoma and received neoadjuvant combinations including 5-fluorouracil, leucovorin, cisplatin, paclitaxel and irinotecan. The authors concluded that the pathological tumour stage was not an independent risk factor for, and did not contribute to the incidence of, surgical complications [34].

### 4.2. Neoadjuvant Chemotherapy and Fitness for Surgery

Systemic anticancer treatment is inherently associated with toxicity. Therefore, the proposed benefit must be considered alongside the potential risk of administration. This is particularly relevant in the context of optimising surgical fitness. To evaluate the impact of neoadjuvant chemotherapy on physical fitness, several investigators utilised cardiopulmonary exercise testing. A systematic review and meta-analysis of the literature explored the impact of neoadjuvant systemic treatment in patients with oesophageal cancer [35]. The authors identified that exercise capacity (*p* = 0.02) and muscle strength (*p* < 0.01) were significantly reduced following neoadjuvant chemotherapy or chemoradiotherapy [35]. With a view to improving preoperative fitness, a non-randomised prospective pilot study investigated the impact of exercise therapy (*n* = 22) on physical fitness following neoadjuvant chemoradiotherapy (NACRT), in comparison with a control group (*n* = 13) [36]. In both groups, objectively measured fitness (oxygen uptake and lactate threshold) was significantly reduced following NACRT. Interestingly, patients in the exercise therapy group demonstrated significantly improved objectively measured fitness in comparison with the control group during the 6-week period following NACRT [36]. Further analysis of the impact of exercise therapy on surgical fitness after neoadjuvant chemotherapy, in the randomised prospective setting, may be beneficial. The PERIOP-OG trial is an RCT designed to investigate this in a cohort of 72 participants with upper gastrointestinal cancers, and results are awaited [37] (NCT03807518).

Next, the relationship between surgical complication risk and targeted forms of SACT will be evaluated.

## 5. Targeted Therapies: Vascular Endothelial Growth Factor Inhibition

Tumorigenesis and metastasis are reliant on effective angiogenesis. Vascular endothelial growth factor (VEGF) is an important mediator of angiogenesis and one that has been established as targetable in anti-cancer therapeutics. Several inhibitory strategies have been investigated and approved for use in multiple cancer types, including both receptor and ligand targeting approaches [38].

### 5.1. Monoclonal Antibody Inhibitors

Strategies that utilise humanised monoclonal antibody agents to disrupt the interaction of VEGF and the VEGFR demonstrated promising evidence of angiogenesis inhibition and restoration of physiological vasculature. This motivated the approval of the first available example, bevacizumab, in patients with metastatic colorectal cancer [39]. Since then, the use of bevacizumab as monotherapy or in combination with chemotherapy or tyrosine kinase inhibitors (TKIs) has been extended to multiple cancer types, including breast and non-small-cell lung cancer [40].

Alternative monoclonal antibody approaches targeted against the VEGFR-2 receptor, such as ramucirumab, form part of the second-line management of gastric and gastroesophageal cancers, whether in isolation or as an adjunct to other interventions [39]. An additional mechanism of VEGF signalling inhibition involves the use of fusion proteins as decoy receptors, or VEGF traps. Aflibercept is an example utilised clinically in the management of wet age-related macular degeneration and metastatic colorectal cancer [41,42].

The bevacizumab expanded access trial (BEAT) was an observational study investigating the safety and efficacy of bevacizumab in 1914 patients with metastatic colorectal cancer [43]. All patients received the physician’s choice of chemotherapy in combination with bevacizumab. Surgical intervention with curative intent was performed in 225 patients, with most participants receiving medical treatment alone. Overall, adverse events relating to bleeding, wound-healing, arterial thromboembolic events or GI perforation occurred in 31%, 4%, 2% and 2% of patients, respectively [43]. Grade 3 or 4 adverse events occurred relatively rarely, with bleeding and GI perforation observed in 3% and 2% of patients, respectively [43]. Serious wound-healing complications and arterial thromboembolic events each occurred in 1% of patients overall [43]. The incidence of adverse events was also analysed specifically among those who underwent surgical intervention [43]. Wound-healing complications occurred in 5% of these patients, with 2% experiencing grade 3 or 4 AEs [43]. Bleeding AEs were reported in 32% of patients; however, 72% of these were epistaxis-related [43]. These data indicate that although bleeding is observed relatively commonly as an adverse effect of bevacizumab, this is often unrelated to any surgical intervention. Overall, the data presented in BEAT suggest that the rate of bleeding and wound-healing complications is higher, albeit in the order of single percentage points, when management includes a combination of surgical intervention and bevacizumab.

The incidence rates of bevacizumab-related AEs observed in the BEAT study were corroborated by data collected in the bevacizumab regimens’ investigation of treatment effects (BRiTE) study, a large observational cohort study including 1953 patients with metastatic colorectal cancer, receiving bevacizumab [44]. It was found that gastrointestinal perforation and arterial thromboembolic events occurred in ≤2% of patients, with grade 3 or 4 bleeding in 2.2%. Postoperative wound-healing complications occurred in 4.4% of 521 patients who underwent surgery whilst on bevacizumab [44]. The authors observed increased incidence of these adverse events among participants who underwent abdominal surgery or had surgery ≤2 weeks after administration of the last dose of bevacizumab. The median time to event in this study ranged from 3.4 months for GI perforation to 5.1 months for arterial thromboembolic events [44]. These observations are important in clinical practice, as an awareness of adverse event timing enables clinical teams to understand when specific complications are likely to occur. They also emphasise the importance of adequate post-SACT recovery.

A systematic review and meta-analysis of 13 non-randomised studies, including 1431 participants with metastatic colorectal cancer, evaluated the safety of bevacizumab prior to liver metastases resection surgery [45]. The authors reported no significant difference in severe complications, including wound-related, thromboembolic and bleeding events, between patients who received preoperative bevacizumab and those that did not [45]. Importantly, eight of the thirteen included studies reported a mean or median interval between bevacizumab-treatment and surgery of ≥6 weeks [45]. This was not provided in 4 studies and was <6 weeks in two studies [45]. Consequently, the conclusions of this study, albeit reassuring, may not be representative of the safety of preoperative bevacizumab within the recommended interval time.

Gastrointestinal perforation and subsequent peritonitis are important complications associated with bevacizumab and have potentially fatal consequences. Management in this scenario centres around timely recognition and emergency surgical intervention. The most appropriate procedure performed under these circumstances is protective diversion stoma creation, with the intention to facilitate successful wound and anastomosis healing [46].

A further effort to demystify the relationship between bevacizumab and surgical wound-healing complications was demonstrated by two randomised studies (AVF 2192 g and AVF 2107 g). The resulting data were pooled and analysed by Scappaticci et al. [47]. The included studies randomised patients to receive bevacizumab in combination with chemotherapy (5-FU/leucovorin or irinotecan/5-FU/leucovorin) or chemotherapy plus placebo (control). The subsequent retrospective analysis of the pooled data separated the 1132 participants into two groups according to whether they underwent surgical intervention prior to starting bevacizumab or during treatment with bevacizumab. Surgery performed prior to starting bevacizumab was associated with grade 3 or 4 wound-healing events in 0.5% and 1.3% of patients in the control arm and bevacizumab-treated arm, respectively [47]. Of the patients who had received bevacizumab prior to undergoing surgery, 13% experienced grade 3 or 4 postoperative wound healing complications, versus 3.4% in the control arm [47]. This study provided randomised, prospective evidence of numerically increased incidence of wound-healing complications with bevacizumab versus control (*p* = 0.63), particularly when bevacizumab is given prior to surgery (*p* = 0.28).

### 5.2. Does a Higher Dose of a VEGF Inhibitor Equal Higher Risk?

The Avastin and Docetaxel (AVADO) study was a randomised, phase III, placebo- controlled trial investigating the efficacy and safety of bevacizumab and docetaxel combination in patients with locally recurrent or metastatic breast cancer. Data relating to the timing and severity of postsurgical wound healing and bleeding complications were collected for all patients who underwent surgery. In AVADO, patients received either: bevacizumab 7.5 mg/kg plus docetaxel, bevacizumab 15 mg/kg plus docetaxel or docetaxel plus placebo. In total, 155 patients underwent surgical procedures, with grade 1–2 bleeding events occurring in 31.6% of those receiving 7.5 mg/kg BV (*n* = 57) and 17.3% receiving 15 mg/kg (*n* = 52) [48]. Wound-healing complications and infections occurred in 1.8% and 1.9% of those randomised to the 7.5 mg/kg BV arm and 15 mg/kg BV arm, respectively [48]. The observed difference in AE incidence between the two groups suggests that bevacizumab dose may be unrelated to the risk of surgical complications, which may be of clinical relevance when considering dose reduction as a method of AE risk reduction. Importantly, the time between bevacizumab dosing and surgery ranged from 0 to 126 days in AVADO, with several cases of major surgery taking place within the 6-week recommended period between the last bevacizumab dose and surgery [48]. Future studies aiming to elucidate the importance of bevacizumab dosing and complication risk should, perhaps, seek to stratify participants according to bevacizumab-surgery time interval in order to shed further light on this.

Avastin therapy for advanced breast cancer (ATHENA), a second investigation conducted in the same clinical setting as AVADO, was a single-arm safety evaluation of bevacizumab including 2251 patients. All patients received bevacizumab (10 mg/kg or 15 mg/kg) plus chemotherapy and 672 patients underwent surgical procedures during bevacizumab treatment [48]. Across all grades, bleeding AEs occurred in 13.5% of participants undergoing surgery, 0.9% were considered grade 3 and a single grade 4 bleeding event was reported [48]. The ATHENA study also investigated wound-healing complications in relation to minor (not requiring general anaesthesia or respiratory assistance) and major surgery, separately. Grade 1 and 2 wound-healing complications occurred in 4.7% and 3.2% of patients undergoing minor and major surgery whilst receiving bevacizumab, respectively [48]. Grade 3 and 4 wound-healing complications including impaired healing and wound dehiscence occurred in four cases (1.3%) of major surgery [48]. Minor surgeries were associated with grade 3 and 4 complications including impaired healing, wound infection and abscess, in eight individuals (2.2%) [48]. Interestingly, the ATHENA study observed all-grade wound-related complications more frequently in minor surgery than in major surgery, suggesting that the use of general anaesthesia or respiratory assistance does not influence complication incidence in this context.

The combined analysis of AVADO and ATHENA, by Cortés et al., demonstrated that grade 3 or 4 wound-healing complications occurred in five participants undergoing major surgery [48]. Of these individuals, three underwent surgical intervention at 21–28 days following bevacizumab termination, within the 6-week recommended period of bevacizumab discontinuation prior to surgery [48]. It is, therefore, recommended that, wherever possible, in the elective setting, a sufficient recovery time of ≥6 weeks is allowed between bevacizumab termination and surgical intervention.

### 5.3. Tyrosine Kinase Inhibitors

Angiogenesis suppression, mediated by VEGF TKIs, is a mechanism utilised in the management of multiple cancer types. Although small-molecule TKIs are approved for use by several international regulatory agencies and are generally well tolerated, the ubiquitous expression of tyrosine kinases combined with off-target and pervasive tyrosine kinase (TK) inhibition gives rise to a unique profile of safety considerations.

The spectrum of adverse effects associated with off-target TK inhibition is broad. However, those of specific relevance to patients with cancer undergoing surgical intervention relate to platelet function. Tyrosine kinase inhibitors are heterogeneous in terms of affinity for platelet TKs, and the mechanism by which TKIs disrupt platelet function is, therefore, varied [49]. Molecules including axitinib, sorafenib and sunitinib have been associated with reduced platelet count [50], and it has been demonstrated that sunitinib delays fibrin formation and inhibits thrombus formation [51]. Other TKIs reduce integrin activation, procoagulant activity or platelet aggregation [50]. Given that effective haemostasis, and by extension wound healing, are dependent on adequate platelet count and function, attention must be paid to how these agents may impact the safety of surgical intervention in patients with cancer.

Two phase II investigations of neoadjuvant axitinib for the treatment of clear cell renal cell carcinoma provided data relating to postoperative complications. The first (NCT01263769) recruited 22 patients with locally advanced, non-metastatic disease, who following treatment with axitinib, underwent partial or radical nephrectomy [52]. Adverse event data demonstrated that grade 3B bleeding and grade 2 wound dehiscence occurred in a single patient each [52]. A subsequent phase II study (NAXIVA) conducted in 20 patients with resectable RCC with venous tumour thrombus extension reported no wound-healing complications [53]. This paucity of significant wound-healing complications continues across multiple individual TKIs. A systematic review of surgical complications associated with neoadjuvant systemic therapies, including TKIs included a single study examining the safety of preoperative sorafenib in patients with renal cell carcinoma [54]. In this study, none of the included participants experienced significant complications relating to wound healing or dehiscence [55].

A systematic review of the Stanford Renal Cancer Surgical Database included 87 patients undergoing surgical intervention for advanced RCC; 14 patients received either sunitinib or sorafenib between 1 and 9 weeks prior to surgery. A control group of 73 patients had not received systemic therapy before surgery. The authors did not appreciate any statistically significant increase in perioperative complication rate associated with preoperative TKI use [56]. However, the incidence and severity of intra-operative adhesions was significantly higher among those receiving preoperative TKIs (86%) versus those that did not (58%) (*p* = 0.01) [56]. There is also evidence of increased risk of bowel anastomosis leakage in patients with metastatic or recurrent gastrointestinal stromal tumours who receive TKIs prior to cytoreductive surgery, compared with those who did not (*p* = 0.032). Notably, 85% of patients in the aforementioned study received imatinib, an inhibitor of BCR-ABL, c-KIT, and PDGFRA, as opposed to VEGF/VEGFR [57].

An alternative TKI, lenvatinib, is licensed for use in thyroid, hepatocellular and endometrial carcinoma. A phase III, randomised study of lenvatinib versus placebo for the treatment of progressive thyroid cancer recruited 392 patients, with 261 receiving lenvatinib [58]. In terms of serious adverse events, a single case each of impaired healing (0.4%) and wound dehiscence (0.4%) was reported [58]. A subsequent study of wound healing complications associated with lenvatinib reviewed the FDA Adverse Event Reporting System database for reports of wound-healing complications between 2015 and 2017 [59]. The case series included nine cases of impaired healing or wound dehiscence, seven of which involved surgery with lenvatinib administered either before or after surgery [59]. Interestingly, 57% of surgery-related wound-healing complications occurred in patients who had lenvatinib discontinued postoperatively. In this study, the authors observed significant variability in time to delayed wound healing (4–58 days), suggesting that regular postoperative monitoring and vigilant wound assessment are required as part of effective management of patients with cancer receiving TKIs [59].

The evidence presented herein suggests that the incidence of VEGF TKI-associated adverse events, specifically bleeding and wound-healing complications, is relatively low versus monoclonal antibody VEGF inhibitors. This is supported by data presented in a systematic review and meta-analysis of the efficacy and safety of TKIs in combination with bevacizumab compared with TKI monotherapy. Grade ≥3 adverse events, including haemorrhage, were reported more commonly among patients receiving combination therapy versus TKI monotherapy (*p* < 0.05) [60].

### 5.4. VEGF Inhibitor Pharmacokinetics

There is disparity in elimination half-life between classes of VEGF inhibitors. Although TKIs are associated with a large volume of distribution and high protein binding, the elimination half-life of agents in this class, albeit varied between molecules, is markedly shorter than that of monoclonal antibody VEGF inhibitors, which is 20 days for bevacizumab (Table 1). Together with elimination half-life, particular attention should be paid to drug specific guidance, as time off treatment requirements are heterogeneous. For example, the elimination half-life of cabozantinib is 99 h; however, a pharmaceutical approval update issued in 2013 suggests that cabozantinib should be stopped for ≥28 days prior to elective surgery.

## 6. Hormonal Therapies and Surgical Risk

Consideration of the safety of hormonal cancer treatment strategies in terms of surgical complication risk is fundamental to improving outcomes in cancers where management incorporates hormonal agents and surgery. This is particularly relevant in patients with breast cancer, where tamoxifen, letrozole and anastrozole regularly form part of neoadjuvant and adjuvant management. A retrospective cohort study including 358 patients with breast cancer who had undergone reconstructive mastectomy investigated the effects of hormonal therapies on surgical complication rates. All participants underwent pedicled transverse rectus abdominis myocutaneous flap reconstruction, 231 participants received hormonal therapy (tamoxifen, anastrozole or letrozole) prior to surgery and 127 did not. The authors did not identify any significant difference in the incidence of hernia, infection, seroma, haematoma or delayed wound healing between the two groups [61]. A significant increase in hernia (*p* = 0.037) and infection (*p* = 0.013) incidence was observed among patients who received letrozole, specifically, versus those who did not receive hormonal treatment [61].

## 7. Surgical Complications in Patients Receiving Radionuclide Therapy

Whilst the relationship between surgical complications and external beam radiation therapy has been evaluated comprehensively in the literature, that of systemic radionuclide therapy is seldom investigated. A small case-matched analysis including 20 patients with metastatic or locally advanced pancreatic neuroendocrine neoplasms compared surgical resection outcomes in two groups of participants; one where patients received neoadjuvant peptide receptor radionuclide therapy (PRRT), and another where upfront surgery was performed. The rate of overall complications was 45% and 60% in those that received neoadjuvant PRRT and those that did not, respectively (*p* = 0.342). Interestingly, the authors observed a significantly reduced rate of pancreatic fistula development in patients that received PRRT versus those that underwent upfront surgery (*p* = 0.011) [62].

In the following section, the safety of immunotherapeutic agents, in terms of surgical outcomes, will be discussed.

## 8. Immunotherapy

The therapeutic armoury available to oncologists was revolutionised in recent years by the introduction of immune checkpoint inhibitors designed to manipulate the immune system to impede cancer cell evasion mechanisms, potentiate immune-cell-mediated response and disrupt carcinogenesis.

Alongside the pioneering mechanism of action of immune checkpoint inhibitors is a novel dossier of immune-related toxicities. Unsurprisingly, adverse events associated with these interventions are multi-system in nature but most commonly affect the gastrointestinal system, liver, skin and endocrine system [63]. Management of these toxicities emanates from accurate diagnosis and grading of adverse events, appropriate clinical investigation and immunosuppression, followed-up with surveillance of any immunosuppressive management after 72 h [64]. Early involvement of relevant specialist physicians is often necessary for serious or complex adverse events. A scenario where early escalation is important is when patients on immune checkpoint inhibitor (ICI) therapy present with fatigue or other vague symptomatology. In these circumstances, it is important that hypophysitis is not overlooked as a consequence of the expected adverse effects of SACT. The European Society of Medical Oncology recommends involvement of an endocrinology specialist, even at grade 1 severity to enable identification and management of hypophysitis [64].

In terms of surgical complication risk with ICIs, the prospective evidence base is limited. However, a retrospective case review of 132 patients with head and neck cancers undergoing reconstructive surgery analysed the incidence of surgical complications associated with immunotherapy [65]. Eighty-two participants were treated with immunotherapy preoperatively, most commonly with pembrolizumab. A total of 89 participants received postoperative immunotherapy and 33 participants received both pre- and post-operative immunotherapy. The likelihood of major complications was increased among those that received preoperative immunotherapy (OR 3.7, *p* = 0.04), and patients in this group required treatment of wound complications significantly more often (OR 2.9, *p* = 0.008) [65].

Aside from wound-related complications, the adverse effects of immunotherapy relating to the gastrointestinal system must be considered when evaluating a patient with cancer for surgical intervention. In a large-scale database analysis based in the United States of America (USA), ICI-induced colitis occurred in 3.6% of patients [66]. A further study investigating outcomes in 39 cases of anti-CTLA4-associated enterocolitis determined that 15% of patients sustained perforation or toxic megacolon [67].

In comparison with neoadjuvant chemotherapy or upfront surgery, there is reassuring evidence supporting the safety and efficacy of surgical resection following neoadjuvant immunotherapy in the setting of advanced oesophageal cancer. A propensity score-matched study, investigating the impact of neoadjuvant immunotherapy and chemotherapy (nICT) on surgical safety, found that postoperative complication rates in the nICT group were comparable to the upfront surgery group [68]. Although the incidence of postoperative pneumonia was significantly increased in the nICT group, neither postoperative hospital stay duration nor 30-day readmission rates were significantly increased among the participants in this group [68].

## 9. Surgical Considerations in the Elective Setting

The impact of any proposed surgical intervention on patients receiving SACT varies according to the type of therapy and the treatment interval. In the elective setting, several recommendations can be made according to the specific treatment agent in use, and they are outlined below.

The literature suggests that patients receiving neoadjuvant chemotherapy are not at a significantly increased risk of surgical complications following an interval of chemotherapy termination prior to surgery (commonly 4–6 weeks). However, in the absence of consensus over a suitable period of recovery between chemotherapy and surgical intervention, a cautious approach should be adopted. Part of this approach involves clinical assessment of blood parameters in order to identify actionable abnormalities such as thrombocytopenia or neutropenia. Effective management of any abnormalities at this stage, either through transfusion, use of CSFs or extended recovery time, may aid risk mitigation. The relevant guidance presented herein should be used to direct these treatment decisions.

Those receiving monoclonal antibody therapies including bevacizumab are more likely to experience surgical complications. Therefore, patients should have treatment terminated approximately 6 weeks prior to surgical intervention. There is also evidence supporting the need for postoperative treatment adjustment in that bevacizumab should not be restarted for at least 28 days postoperatively [69].

Whilst it was established previously that the elimination time of TKIs is often significantly shorter than monoclonal antibody molecules, a period of recovery should be allowed between treatment termination and surgery. Importantly, the exact interval in these cases is variable and TKI-dependent (Table 1). Specific recommendations relating to the duration of this recovery period should be sought via pharmaceutical approval documentation.

Although the understanding of immunotherapy-related adverse events continues to develop, it is recommended that surgical teams acquire an awareness of the associated toxicities, with particular emphasis on those that may complicate, or increase the risk of, surgical interventions.

## 10. Surgical Considerations in the Emergency Setting

It is acknowledged that, in an emergency situation, it may be practically or clinically inappropriate to withhold medical interventions in accordance with the recommendations that apply to the elective setting. However, an understanding of the biology and pharmacokinetics of specific treatment types, and timely involvement of the responsible oncologist, may facilitate mitigation of surgical risk as far as possible. An understanding of the risks associated with SACT will enable clinicians to obtain consent from patients in a way that reflects the possible increase in complication risk and enables balanced consideration of the proposed benefit. It is also important that any comorbidities such as hypertension and proteinuria, and risk factors for venous thromboembolism and gastrointestinal perforation are recognised and managed accordingly.

As was mentioned previously, the use of CSFs may be appropriate for the treatment of neutropenia under certain urgent circumstances, where time constraints permit.

In terms of optimising surgical planning to mitigate risk among patients in receipt of SACT, there are insufficient data available to make recommendations on specific approaches or surgical techniques under these circumstances. A laparoscopic approach has been shown to be associated with reduced surgical site infections in routine elective practice [70,71] and should be considered in this group where the patient’s physiology and pathology allow. Given the potentially significant sequelae of infective complications in this cohort of patients, surgeons must utilise their expertise and judgement with respect to the appropriateness of anastomosis and use of surgical drains.

## 11. Patient Participation in Clinical Trials: Expedited Safety Reporting

As clinical trial participation becomes increasingly integrated with care provision, it is possible that patients presenting to primary or secondary care as a result of serious adverse events are participating in an ongoing clinical trial.

The guidelines for good clinical practice stipulate that all serious adverse events are reported in a timely manner to the sponsor of the clinical study and any relevant regulatory authorities. In order to adhere to these requirements, the responsible oncologist must be informed of any admission to the emergency department or planned surgical intervention, with the details of the presenting complaint, diagnosis and proposed management included.

## 12. Conclusions and Future Directions

As systemic anticancer therapy continues to evolve and the role of surgical intervention in cancer management remains crucial in improving patient outcomes, a comprehensive understanding of the safety concerns associated with contemporary anticancer agents is essential if surgical risk attenuation is to be achieved. The available literature presented here indicate that the incidence of SACT-related surgical complications and the magnitude of safety concern varies between therapy classes and is often highly situation dependent. Where SACT adverse effects can be actioned to minimise surgical risk, appropriate guidance should be sought and incorporated into management planning. Further investigations aiming to identify the optimum duration of agent-specific treatment cessation intervals between SACT and surgery may be of value in minimising surgical complications. As gene therapy progresses, the safety implications of these agents from a surgical perspective should be evaluated, as currently there is a sparsity of literature in this regard. Finally, a more detailed understanding of the safety profiles of immunotherapeutic agents in this context would be beneficial, especially given the likely permeation of these modalities into the treatment regimens of further cancer types in future.

## Figures and Tables

**Table 1:** Selected vascular endothelial growth factor-targeted therapies with associated half-life timing and recommended treatment termination time before and after surgery. Abbreviations: mAb: monoclonal antibodies; VEGF: vascular endothelial growth factor; VEGFR: vascular endothelial growth factor receptor; TKI: tyrosine kinase inhibitor, PDGFR: platelet derived growth factor receptor, FLT; fms-like tyrosine kinase 3, CSFR; colony-stimulating factor-1 receptor. * Interval recommendations according to summary of product characteristics documentation provided by European Medicine Agency or U.S. Food and Drug Administration.

Molecule	Mechanism	Half-Life	Interval Recommendation *
Bevacizumab	mAb VEGF-A inhibition	20 days	Discontinue for ≥28 days prior to and after surgery
Ramucirumab	mAb VEGFR2 antagonist	13.4 days	Discontinue for ≥4 weeks prior to surgery and after surgery if there are wound healing complications
Aflibercept	Protein decoy for VEGF A-D and PIGF	11 days	Withhold within the previous or next 28 days in the event of a performed or planned intraocular surgery
Axitinib	TKI of VEGFR-1-3	2.5–6.1 h	Discontinue ≥ 24 h prior to scheduled surgery
Sorafenib	TKI of VEGFR, PDGFR, c-Kit and RET	25–48 h	No specific interval recommendation
Sunitinib	Multi-targeted TKI including PDGFRα, PDGFRβ, VEGFR1, VEGFR2, VEGFR3	40–60 h	Discontinue for ≥3 weeks prior to surgery and for ≥2 weeks after major surgery
Imatinib	Multi-targeted TKI including BCR-ABL, c-KIT, and PDGFRA	20 h	No specific interval recommendation
Lenvatinib	Multi-targeted TKI including VEGFR-1-3 and FGFR-1-4	28 h	No specific interval recommendation
Cabozantinib	Multi-targeted TKI including VEGFR-1-3	99 h	Discontinue for ≥28 days prior to scheduled surgery
Pazopanib	Multi-targeted TKI including VEGFR1-3, PDGFRβ and FGFR1	21–51 h	Discontinue for ≥7 days prior to scheduled surgery
Regorafenib	Multi-targeted TKI including VEGFR1-3, PDGFR and FGFR1-2	20–30 h	No specific interval recommendation
